# Supplementary material for: Assessment of Dental Fear and Anxiety Tools for Children: A Review
Source: Healthcare (Basel). 2025 Oct 15;13(20):2597. doi: 10.3390/healthcare13202597 (PMC12564910; doi:10.3390/healthcare13202597)
Supplement: Supplementary file 1 [file healthcare-13-02597-s001.zip › healthcare-3881989-supplementary.pdf]

Supplemental Table 1. Comparison of Dental Anxiety Scales Using PRO Checklist

Criteria	C-DAS	MDAS	MCDAS	MCDASf	VPS	CFSS-DS	DFSS-SF	SFP	SFP-R	FIS	ACDAS	CEDAM	CEDAM-8	VCARS
**conceptual model**														
1. Has the PRO construct to be measured been specifically defined?	1	1	1	1	1	1	1	1	1	1	1	1	1	1
2. Has the intended respondent population been described?	1	1	1	1	1	1	1	1	1	1	1	1	1	1
3. Does the conceptual model address whether a single construct/scale or multiple subscales are expected?	1	1	1	1	1	1	1	1	1	1	1	1	1	1
**Content Validity**														
4. Is there evidence that members of the intended respondent population were involved in the PRO measure's development?	1	1	1	1	1	1	1	1	1	1	1	1	1	0
5. Is there evidence that content experts were involved in the PRO measure's development?	1	1	1	1	1	1	1	1	1	1	1	1	1	1
6. Is there a description of the methodology by which items/questions were determined (e.g., focus groups, interviews)?	1	1	1	1	1	1	1	1	1	1	1	1	1	1
**Reliability**														
7. Is there evidence that the PRO measure's reliability was tested (e.g., test-retest, internal consistency)?	1	1	1	1	1	1	1	1	1	1	1	1	1	1
8. Are reported indices of reliability adequate (e.g., ideal: $r \geq 0.80$; adequate: $r \geq 0.70$; or otherwise justified)?	1	1	1	1	1	1	1	1	1	1	1	1	1	1
9. Is there reported quantitative justification that single scale or multiple subscales exist in the PRO measure (e.g., factor analysis, item response theory)?	0	0	0	0	0	0	0	0	0	0	0	0	0	0
**Construct Validity**														
10. Are there findings supporting expected associations with existing PRO measures or with other relevant data?	1	1	1	1	1	1	1	1	1	1	1	1	1	1
11. Are there findings supporting expected differences in scores between relevant known groups?	1	1	1	1	1	1	1	1	1	1	1	1	1	1

12. Is the PRO measure intended to measure change over time? If YES, is there evidence of both test-retest reliability AND responsiveness to change? Otherwise, award 1 point if there is an explicit statement that the PRO measure is NOT intended to measure change over time.	0	0	0	0	0	0	0	0	0	0	0	1	1	0
**Scoring & Interpretation**														
13. Is there documentation how to score the PRO measure (e.g., scoring method such as summing or an algorithm)?	1	1	1	1	1	1	1	1	1	1	1	1	1	1
14. Has a plan for managing and/or interpreting missing responses been described (i.e., how to score incomplete surveys)?	0	0	0	0	0	0	0	0	0	0	0	0	0	0
15. Is information provided about how to interpret the PRO measure scores (e.g., scaling/anchors, what high and low scores represent, normative data, and/or a definition of severity [mild → severe])?	1	1	1	1	1	1	1	1	1	1	1	1	1	1
**Respondent Burden & Presentation**														
16. Is the time to complete reported and reasonable? OR, if it is NOT reported, is the number of questions appropriate for the intended application?	1	1	1	1	1	1	1	1	1	1	1	1	1	1
17. Is there a description of the literacy level of the PRO measure?	0	0	1	1	1	1	1	1	1	1	1	1	1	1
18. Is the entire PRO measure available for public viewing (e.g., published with the citation, or information provided about how to access a copy)?	1	1	1	1	1	1	1	0	1	1	1	1	1	1
**Total**	14	14	15	15	15	15	15	14	15	15	15	16	16	14
